# Global Analysis of Serine/Threonine and Tyrosine Protein Phosphatase Catalytic Subunit Genes in *Neurospora crassa* Reveals Interplay Between Phosphatases and the p38 Mitogen-Activated Protein Kinase

**DOI:** 10.1534/g3.113.008813

**Published:** 2013-12-17

**Authors:** Arit Ghosh, Jacqueline A. Servin, Gyungsoon Park, Katherine A. Borkovich

**Affiliations:** * Department of Plant Pathology and Microbiology, University of California, Riverside, California 92521; † Graduate Program in Genetics, Genomics, and Bioinformatics, University of California, Riverside, California 92521; ‡ Institute for Integrative Genome Biology, University of California, Riverside, California 92521

**Keywords:** filamentous fungi, protein phosphorylation, functional genomics, serine-threonine protein phosphatases, tyrosine protein phosphatases

## Abstract

Protein phosphatases are integral components of the cellular signaling machinery in eukaryotes, regulating diverse aspects of growth and development. The genome of the filamentous fungus and model organism *Neurospora crassa* encodes catalytic subunits for 30 protein phosphatase genes. In this study, we have characterized 24 viable *N. crassa* phosphatase catalytic subunit knockout mutants for phenotypes during growth, asexual development, and sexual development. We found that 91% of the mutants had defects in at least one of these traits, whereas 29% possessed phenotypes in all three. Chemical sensitivity screens were conducted to reveal additional phenotypes for the mutants. This resulted in the identification of at least one chemical sensitivity phenotype for 17 phosphatase knockout mutants, including novel chemical sensitivities for two phosphatase mutants lacking a growth or developmental phenotype. Hence, chemical sensitivity or growth/developmental phenotype was observed for all 24 viable mutants. We investigated p38 mitogen-activated protein kinase (MAPK) phosphorylation profiles in the phosphatase mutants and identified nine potential candidates for regulators of the p38 MAPK. We demonstrated that the PP2C class phosphatase *pph-8* (NCU04600) is an important regulator of female sexual development in *N. crassa*. In addition, we showed that the Δ*csp-6* (ΔNCU08380) mutant exhibits a phenotype similar to the previously identified conidial separation mutants, Δ*csp-1* and Δ*csp-2*, that lack transcription factors important for regulation of conidiation and the circadian clock.

Phosphorylation of three amino acids—serine, threonine, and tyrosine—regulates myriad biological reactions in eukaryotic cells (Bauman and Scott 2002). Such regulatory cascades involve a cycle of phosphorylation via kinases and subsequent removal of the phosphate groups by phosphatases. A proper balance between kinases and phosphatases is essential for the maintenance of cell homeostasis. Because of their importance to cellular processes, kinases and phosphatases are among the most extensively studied enzymes (Cohen 2001; Hunter 1995). Phosphatases have been classified according to sequence homology, structural characteristics, and substrate specificity (Pao et al 2007; Shi 2009). Based on such properties, there are two major families of phosphatases: serine/threonine (S/T) protein phosphatases and protein tyrosine phosphatases. In general, protein phosphatases perform dephosphorylation in a mechanism involving nucleophilic attack on the phosphate ester moiety of the substrate (Sanvoisin and Gani 2001; Williams 2004). S/T phosphatases initiate the nucleophilic attack by means of a metal-activated water molecule in the catalytic groove, whereas protein tyrosine phosphatases use a catalytic cysteine residue as the nucleophile (McConnell and Wadzinski 2009).

S/T phosphatases are further classified into three main subfamilies, phosphoprotein phosphatases (PPPs), metal-dependent protein phosphatases (PPMs), and aspartate-based protein phosphatases, comprising the transcription factor IIF–interacting C-terminal domain phosphatase (FCP/SCP) and haloacid dehalogenase (HAD) classes (Shi 2009; Zhang *et al.* 2010). Protein tyrosine phosphatases are subdivided into classical protein-tyrosine phosphatases (PTPs), dual-specificity phosphatases (DSPs), low-molecular-weight phosphatases (LMW-PTP), and CDC25 class phosphatases (Andersen *et al.* 2001; Moorhead *et al.* 2007; Pao *et al.* 2007).

The PPP subfamily of S/T phosphatases has been implicated in a broad range of cellular processes, such as metabolism, cytoskeletal rearrangements, base excision repair, mitotic entry, and regulation of membrane receptors and ion channels (Burgess *et al.* 2010; Herzig and Neumann 2000; Lorca *et al.* 2010; Lu *et al.* 2004). Differences in relative inhibition by okadaic acid (Bialojan and Takai 1988) have led to subdivision of the PPP subfamily into PP2A, PP2B (calcineurin-A), and PP5 classes. PP2A is a highly conserved and ubiquitous protein phosphatase class, accounting for as much as 1% of total cellular proteins (Cohen 1990). The PP2A core enzyme consists of the PP2A catalytic or C subunit and the scaffolding A subunit [huntingtin-elongation-A subunit of PP2A TOR (HEAT) motifs] existing as a heterodimer that associates with the regulatory B subunit to constitute a heterotrimeric holoenzyme complex (Cho and Xu 2007; Xing *et al.* 2006; Xu *et al.* 2006). In mammals, PP2A class phosphatases dephosphorylate the microtubule-associated protein Tau and modulate mitogen-activated protein kinase (MAPK) signaling pathways by dephosphorylation of the component kinases (Janssens and Goris 2001). The PP2A class protein phosphatases PPP-1 and PPH-1 (catalytic subunits encoded by NCU00043; *ppp-1* and NCU06630; *pph-1*) are important regulators of the circadian clock in *Neurospora* (Yang *et al.* 2004). Previous studies were successful in generating viable partial Repeat-Induced Point (RIP) mutants for *ppp-1*, but not for *pph-1* (Yang *et al.* 2004). Although PPP-1 has been shown to influence the circadian clock by regulating the stability of the clock protein FRQ via dephosphorylation, *in vitro* studies demonstrated that the PP2A class holoenzyme dephosphorylates FRQ via the PPH-1 phosphatase but does not affect FRQ stability (Yang *et al.* 2004). Furthermore, dephosphorylation of the WCC (transcriptional activator of FRQ) has been shown to be dependent on the PP2A regulatory subunit RGB-1, leading to activation of WC-1 (Schafmeier *et al.* 2005).

The PPP class phosphatase calcineurin-A is a calcium-calmodulin–dependent enzyme (Bandyopadhyay *et al.* 2002; Klee *et al.* 1998). In animals, calcineurin dephosphorylates and activates the transcription factor NFATc, leading to T-cell differentiation via interleukin-2 expression (Crabtree 1999; Rusnak and Mertz 2000). Calcineurin is an essential gene involved in hyphal growth and maintenance of Ca^+2^ gradients in *Neurospora crassa* (Prokisch *et al.* 1997). The calcineurin holoenzyme is a heterodimer and consists of the large catalytic A subunit and a small regulatory B subunit (Klee *et al.* 1988). In *Saccharomyces cerevisiae*, *cna1* and *cna2* are functionally redundant catalytic subunits for calcineurin and, although *cna1 cna2* double-mutants are viable, they are more sensitive to high levels of sodium, lithium, and other ions in the growth medium (Farcasanu *et al.* 1995; Garrett-Engele *et al.* 1995). Deletion of the *cna-1* homolog is not lethal but results in a weakly growing mutant in *Aspergillus nidulans* (Cyert *et al.* 1991; Feng *et al.* 1991; Son and Osmani 2009). The regulatory subunit of calcineurin is encoded by the *cnb1* gene in *S. cereviseae* and is required for adaptation to pheromone *in vivo* (Cyert and Thorner 1992), whereas in *Neurospora* the *cnb-1* gene is required for normal vegetative growth (Kothe and Free 1998a).

The PPM subfamily of S/T phosphatases consists of PP2C enzymes with well-documented roles in cell-cycle progression (Cheng *et al.* 1999; Leroy *et al.* 2003; Lu and Wang 2008) and tumorgenicity (Ofek *et al.* 2003) in animals and act as negative regulators of the abscisic acid (ABA) signaling pathway in the model plant *Arabidopsis thaliana* (Ma *et al.* 2009). PP2C phosphatases act on a number of MAPK pathways (Arino *et al.* 2011). For example, Ptc1p in *S. cerevisiae* inactivates the high osmolarity glycerol (HOG) pathway by dephosphorylating the Hog1 MAPK (Warmka *et al.* 2001).

The FCP/SCP and HAD phosphatases are an aspartate-based class of S/T phosphatases with a shared DxDxT/V sequence motif. FCP1 is an essential protein phosphatase that dephosphorylates the C-terminal domain (CTD) of the largest subunit of RNA polymerase II (Archambault *et al.* 1998; Kobor *et al.* 1999). The HAD class of protein phosphatases contains important regulators of actin-cytoskeleton dynamics in mammals (Gohla *et al.* 2005; Seifried *et al.* 2013)

The PTP family is distinguished by a signature HC(X_5_)R catalytic motif. These proteins play important roles during meiosis and sporulation in yeast and cell adhesion, metabolism, and immune cell signaling in mammals (Elchebly *et al.* 1999; Mustelin *et al.* 2004; Zhan *et al.* 2000). Classical PTPs can be classified as receptor and nonreceptor PTPs and these phosphatases have functions in cell–substrate and cell–cell adhesion as well as insulin signaling in animals (Stoker 2005). DSPs dephosphorylate phosphotyrosine, phosphoserine, and phosphothreonine residues on substrates (Alonso *et al.* 2004; Tonks 2006). DSPs can be further classified on the basis of the presence (typical) or absence (atypical) of a MAPK-interacting domain, (Huang and Tan 2012; Jeffrey *et al.* 2007). For example, in *Ustilago maydis*, the DSP Rok1 is known to regulate mating and virulence by controlling the phosphorylation of Erk MAPKs Kpp2 and Kpp6 (Di Stasio *et al.* 2009). Among the other classes of PTPs, CDC25-type phosphatases have essential roles in mitotic entry (Gautier *et al.* 1991), whereas the LMW-PTP is less well-understood. In addition to these major classes of PTPs, SSU72 is a unique RNA polymerase II CTD phosphatase that shares high sequence similarity with PTPs (Ganem *et al.* 2003; Zhang *et al.* 2012). Y-phosphatases are a lesser studied class of PTPs that seem to be unique to filamentous fungi. In *A. nidulans*, AN4426 (Son and Osmani 2009) is a Y-phosphatase homologous to SIW14, a tyrosine phosphatase involved in endocytosis in *S. cerevisiae* (Sakumoto *et al.* 2002).

The filamentous fungus *N. crassa* is a model system for investigations of cell growth, development, gene silencing, the circadian clock, and stress responses in eukaryotic cells (Borkovich *et al.* 2004; Davis and Perkins 2002). *N. crassa* possesses 16 S/T phosphatases and 14 PTPs. Among the previously characterized protein phosphatases in *N. crassa*, the PP2A phosphatase *pp2A* (NCU06563) is involved in hyphal growth and cell–cell fusion (Fu *et al.* 2011; Pandey *et al.* 2004; Yatzkan *et al.* 1998), whereas another PP2A phosphatase, *pph-1* (NCU06630), has so far been implicated in hyphal growth (Yang *et al.* 2004; Yatzkan *et al.* 1998). Mutation of the *tangerine/tng* gene (NCU03436), an ortholog of the cell-shape-control protein phosphatase *cpp-1* in *Fusarium verticillioides*, leads to swollen hyphae and hyperbranching at the colony edge (McCluskey *et al.* 2011).

Taking advantage of the publicly available *N. crassa* genome sequence (Galagan *et al.* 2003) and the large-scale gene knockout project for ∼10,000 predicted genes (Park *et al.* 2011a), we have previously investigated the effects of mutating 86 S/T kinase genes in *N. crassa* (Colot *et al.* 2006; Park *et al.* 2011b). To elucidate the functions of protein phosphatases in *N. crassa*, we initiated a systematic analysis of the 30 predicted genes. In this study, we analyzed 24 viable phosphatase mutants for defects in basal growth, asexual development, and sexual development. Chemical sensitivity testing has proven to be a powerful method for identification of phenotypes for gene deletion mutants and genes of unknown function, as evident from previous studies in *S. cerevisiae* (Hillenmeyer *et al.* 2008) and our analysis of protein kinases (Park *et al.* 2011b). Accordingly, we tested the phosphatase mutants for altered sensitivity to several chemical stresses and growth under different nutritional regimens. We also measured phosphorylation of the p38 MAPK (OS-2) in all viable mutants to identify potential phosphatases acting on this pathway in *N. crassa*. The results reveal at least one defect for every phosphatase mutant analyzed, demonstrating the importance of these proteins to *N. crassa* biology. We present evidence linking the PP2C phosphatase *pph-8* (NCU04600) and the HAD family phosphatase *csp-6* (NCU08380) to important aspects of sexual and asexual development in *N. crassa*. We identified several protein phosphatases that influence basal or induced phosphorylation of the OS-2 MAPK.

## Materials and Methods

### *Neurospora crassa* strains and growth conditions

Wild-type strains ORS-SL6a [Fungal Genetics Stock Center (FGSC) 4200; *mat a*] and 74-OR23-IVA (FGSC 2489; *mat A*) and phosphatase mutants produced during the knockout project (Table 1) were obtained from the FGSC (Kansas City, MO). Knockout mutants for three phosphatase genes were not available as either homokaryons or heterokaryons (Table 1). Vegetative growth and asexual development (conidiation) were analyzed using Vogel minimal medium (VM) (Vogel 1956), whereas sexual development was assessed using synthetic crossing medium (SCM) (Westergaard and Mitchell 1947). Conidia used for inoculating cultures were propagated in VM agar flask cultures grown for 3 d at 30° in the dark and for 4 d at 25° in the light. Sorbose-containing medium (FGS) was used for isolation of colonies on plates and for ascospore germination assays (Davis and DeSerres 1970). When indicated, VM was supplemented with hygromycin (Calbiochem, San Diego, CA) at a concentration of 200 μg/ml.

**Table 1:** *Neurospora crassa* phosphatase gene families and summary of phenotypes and p38 MAPK levels

Family*^a^*	Subfamily*^b^*	Class/Domain*^c^*	NCU*^d^*	*N. crassa* Gene*^e^*	*S. cerevisiae Homolog**^f^*	Phenotype Summary				Chemical Sensitivity*^j^*/Nutrition*^k^*	Phospho-p38 MAPK*^l^*
						Inviable	Linear Growth*^g^*	Asexual Development*^h^*	Sexual Development*^i^*		
S/T	PPP	PP2Ac	00043	*ppp-1/ pph-3*	*GLC7*	X	—*^m^*	—	—	—	—
S/T	PPP	PP2Ac	03436	*tng*	*SIT4*		R	AH, C	PP, P, A	N/S*^n^*	
S/T	PPP	PP2Ac	06563	*pp2A*	*PPG1*			C	PP, P, A	SC, S, B, M, FL, T,	B
S/T	PPP	PP2Ac	07489	*pzl-1*	*PPZ1*		R	AH, C	PP	SC, S, B, M, T, F, YE	
S/T	PPP	PP2Ac	08301	*pph-4*	*PPH3*		R	AH	PP, A	C, M, T, YE, A	
S/T	PPP	PP2Ac	06630	*pph-1*	*PPH21*	X	—	—	—	—	—
S/T	PPP	PP2Bc	03804	*cna-1/ pph-2*	*CMP2*	X	—	—	—	—	—
S/T	PPP	PP5c	01433	*ppt-1*	*PPT1*		I			T	
S/T	PPM	PP2Cc	00958	*pph-7*	*PTC7/AZR1*					FL	
S/T	PPM	PP2Cc	01767	*pph-5*	*PTC5*		R	AH*		T	
S/T	PPM	PP2Cc	03495	*pph-6*	*PTC6*		I			T, F	
S/T	PPM	PP2Cc	04600	*pph-8*	*PTC2*		R	AH, C	PP, P, A	N/S	B
S/T	PPM	PP2Cc	00434	*pph-9*	*PTC1*	na*^o^*	na	na	na	na	na
S/T	Asp-Based	HAD	08948	*pph-11*	*PSR1*		R	AH, C	PP, P, A	N/S	B
S/T	Asp-Based	HAD	08380	*csp-6*	*PSR2*		R	AH, C	PP, P, A	SC, B, FL, T, F	
S/T	Asp-Based	FCP/SCP	09300	*fcp-1*	*FCP1*	X	—	—	—	—	—
PTP	Classical	PTPc	02257	*pty-2*	*PTP1*		I			T, F	
PTP	Classical	PTPc	05364	*pty-3*	*PTP2/PTP3*				PP, P, A	S	B, I
PTP	Dual-specificity	DSPc	03246	*cdc-14*	*CDC14*		R	AH*, C	A	S, B, F	
PTP	Dual-specificity	DSPc	03426	*dsp-1*	*PPS1*			AH*		T	
PTP	Dual-specificity	DSPc	06252	*dsp-2*	*MSG5*			AH, C	PP, P, A	S, T, A	
PTP	Dual-specificity	DSPc	06330	*dsp-3*	*MSG5*		R		PP, P, A		
PTP	Dual-specificity	DSPc	08158	*dsp-4*	*YVH1*				PP, A		
PTP	Dual-specificity	DSPc	05049	*dsp-5*	*SDP1*	na	na	na	na	na	na
PTP	LM-PTP	LMWPc	09841	*pty-4*	*LTP1*				PP	M, F	B
PTP	CDC-25 type	CDC25	02496	*div-12*	*MIH1*				PP, P, A	F	B, I
PTP	CDC-25 type	CDC25	06966	*pty-1*	*YCH1*			C			B
PTP	SSU72	SSU72	03114	*pph-10*	*SSU72*			AH*	PP, P, A	C	B
PTP	—	Y-phosphatase 3	01010	*pty-5*	*—*			C			
PTP	—	Y-phosphatase 2	03333	*pty-6*	*SIW14*					F	

a Family abbreviations: S/T, serine/threonine; PTP, protein tyrosine phosphatase.

b Subfamily abbreviations: PPP, phosphoprotein phosphatase; PPM, Mg^2+^ or Mn^2+^-dependent protein phosphatase; Asp-based, aspartate-based phosphatase; LMW-PTP, low-molecular-weight protein tyrosine phosphatase; CDC25 type, cell division cycle 25 type; SSU72, C-terminal domain RNA Pol II phosphatase.

c Class/domain abbreviations: PP2Ac, protein phosphatase 2 A catalytic subunit; PP2Bc, protein phosphatase 2 B catalytic subunit; PP5 catalytic subunit, protein phosphatase 5 catalytic subunit; PP2Cc, protein phosphatase 2C catalytic subunit; HAD, haloacid dehalogenase; FCP/SCP, transcription factor IIF–interacting C-terminal domain phosphatase 1/ small C-terminal domain phosphatase; PTPc, protein tyrosine phosphatase catalytic subunit; DSPc, dual-specificity phosphatase catalytic subunit; LMWPc, low-molecular-weight phosphatase catalytic subunit; CDC25, cell division cycle; SSU72, C-terminal domain RNA polymerase II phosphatase; Y-phosphatase 3, tyrosine phosphatase 3; Y-phosphatase 2, tyrosine phosphatase 2.

d Based on version 5 annotation of the Broad Institute’s *Neurospora crassa* database (http://www.broadinstitute.org/annotation/genome/neurospora/MultiHome.html).

e Phosphatase gene names are consistent with the *Neurospora* e-Compendium Project at Leeds University (http://bmbpcu36.leeds.ac.uk/~gen6ar/newgenelist/genes/gene_list.html). All other gene names were given during this study in accordance with the e-Compendium system.

f Yeast orthologs were obtained from literature or blastp search and are consistent the Saccharomyces Genome Database (http://www.yeastgenome.org/).

g R, reduced growth; I, increased growth (significance testing performed with Student *t* test, paired, two-tailed; p<0.05, *p<0.1).

h Asexual phenotypes are depicted by phenotypes in aerial hyphae (AH) or conidial development (C).

i Sexual phenotypes are depicted by their occurrence during protoperithecial (PP), perithecial (P), or ascospore (A) development.

j Chemical sensitivity phenotypes are represented based on the sensitivity or resistance to sodium chloride (SC), sorbitol (S), cytochalasin A (C), benomyl (B), tert-butyl hydroperoxide (T), menadione (M), FK506 (F), fludioxonil (FL), and yeast extract (YE).

k Nutritional phenotypes are represented by increased growth on Avicel (A) as compared to wild-type.

l p38 MAPK levels are represented relative to wild-type levels as elevated basal (B) or elevated induced (I).

m Phenotypic analysis could not be performed because of inviability of the knockout mutants.

n N/S, mutant strain was not analyzed for chemical screening because of poor growth compared to wild-type.

o na, mutant not available.

In this study, the gene names for *N. crassa* phosphatases were taken from the literature (the e-compendium at Leeds University; http://bmbpcu36.leeds.ac.uk/~gen6ar/newgenelist/genes/gene_list.htm) or were assigned a name (Table 1).

### Purification of homokaryotic phosphatase mutants from heterokaryons

Because *N. crassa* is multinucleate, primary transformants are often heterokaryons, with both mutant and wild-type nuclei (Dev and Maheshwari 2002; Paietta and Marzluf 1985; Park *et al.* 2011a). Therefore, transformants were crossed to wild-type to purify homokaryotic meiotic progeny for the knockout project (Colot *et al.* 2006; Park *et al.* 2011a). Using the aforementioned method, one phosphatase cassette did not yield transformants (Δ*pph-9*; NCU000434) and viable ascospores could not be isolated for four phosphatase mutants: Δ*ppp-1* (NCU00043); Δ*cna-1* (NCU03804); Δ*pph-1* (NCU06630); and Δ*div-12* (NCU02496). Homokaryotic mutants for Δ*div-12* (NCU02496) were purified after serial plating of conidia. Macroconidia were plated on FGS-hygromycin plates and incubated in the dark at 30°. The next day, one colony was picked and transferred to a VM-hygromycin agar slant and cultured for 5 d. Macroconidia were isolated from this slant and plated on a FGS-hygromycin plate; after incubation, a colony was transferred onto a fresh VM-hygromycin slant. These steps were repeated twice. Diagnostic PCR with gene-specific and *hph* primers (Supporting Information, Table S2) was used to test for the absence of the open-reading frame of the respective deleted gene (with wild-type as a positive control) and the simultaneous presence of the *hph* cassette in the purified strains (Colot *et al.* 2006). These experiments confirmed that the purified strains were homokaryons.

### Analysis of growth and morphological and developmental phenotypes

The 24 viable *N. crassa* phosphatase knockout mutants (Table 1) were analyzed for phenotypes using methods reported previously (Turner 2011), with some modifications. Linear growth rates for the mutants (Table S1) were measured on VM at 25° in the dark using race tubes (Turner 2011). Mutants were grown on VM plates for 24 hr and hyphae at the colony edge were photographed using an Olympus SZX9 stereomicroscope with a C-4040 digital camera (Olympus, Lake Success, NY). VM slant tubes were inoculated with the mutant strains and grown for 3 d in the dark at 30°, for 4 d under constant light at 25°, and then scored for conidial production (Table S1). Aerial hyphal extension was measured in 2 ml VM (standing) liquid cultures. These cultures were inoculated at the liquid surface and incubated statically at 25° (in the dark) for 96 hr. The total height of aerial hyphae was measured in millimeters (Table S1). Data were subsequently tested for significance using Student *t* test (paired, two-tailed, independent means).

For analysis of female sexual fertility, strains were inoculated onto SCM slants and incubated under constant light for 7 d to 8 d at 25°. Cultures were scored for protoperithecia formation and then fertilized with conidia of the opposite mating type. Subsequent perithecia formation and ascospore development were scored 1 wk and 2 wk after fertilization, respectively. All scoring for female sexual fertility analysis was performed using the SZX9 stereomicroscope (Olympus). For visualizing unregulated protoperithecial formation in the Δ*pph-8* mutant (NCU04600), the strain was inoculated on VM and SCM agar plates and incubated under constant light or dark at 25° as indicated in Figure 3. A wild-type strain (FGSC 2489; *mat A*) was used as a control. Photographs were taken using the SZX9 stereomicroscope with a C-4040 digital camera (Olympus) at 5 d and 7 d after inoculation and 2 d after fertilization.

Conidial separation was investigated in wild-type (FGSC 4200; *mat a*), Δ*csp-1* (NCU02713, FGSC 2555), Δ*csp-2* (NCU06095, FGSC 2522), and Δ*csp-6* (NCU08380) strains. Conidia were propagated by culturing strains in VM agar flasks for 3 d in the dark at 30° and for 4 d in the light at 25°. A small amount of conidia was withdrawn from the flask, suspended in 50 μl of sterile liquid VM, and 50 μl of calcofluor white (Eng Scientific, Clifton, NJ) was added to the suspension. A volume of 20 μl was placed on a glass slide and covered with a cover slip. Conidia were visualized using differential interference (DIC) microscopy with an IX71 inverted microscope (Olympus America, Center Valley, PA) using a 60× oil immersion objective. X-Cite 120PC Q (Lumen Dynamics, Ontario, Canada) was used as the fluorescence microscope light source with a DAPI filter cube on the microscope. Photographs were taken using a QIClick digital CCD camera (QImaging, Surrey, British Columbia, Canada).

### Chemical sensitivity assays and nutritional phenotypes

Chemical sensitivity assays were restricted to viable phosphatase knockout mutants with growth rates at least 50% of the wild-type strain on VM as shown in Table S1 (Park *et al.* 2011b). The mutants were screened for responses to a variety of chemicals at concentrations that inhibited wild-type growth by ∼50–60% (Table S1). The chemicals included sodium chloride (0.35 M; EMD Chemicals, Gibbstown, NJ), sorbitol (0.8 M; Sigma, St. Louis, MO), cytochalasin A (40 ng/ml; Sigma), benomyl (92 ng/ml; Fluka, St. Louis, MO), *tert*-butyl hydroperoxide (*t*-BuOOH; 0.13 mM; Sigma), Menadione (100 μM; M5750; Sigma), FK-506 (50 ng/ml; LC Laboratories, Woburn, MA), and fludioxonil (2.75 ng/ml; a gift from Frank Wong and Allison Tally). Phosphatase mutants were also analyzed for nutritional phenotypes, including growth on VM supplemented with 2% yeast extract and utilization of crystalline cellulose (Avicel; PH-101; Sigma) as a carbon source (Haas *et al.* 1952; St. Lawrence *et al.* 1964; Znameroski *et al.* 2012). VM plates (60 mm × 15 mm) were supplemented with the respective chemicals and one edge of the plate was inoculated and radial colony growth was measured after 20–22 hr at 30°. A VM plate lacking chemical was used as a control for each of the tested strains. The percentage growth was calculated by dividing the radius with chemical by the radius in the absence of chemical for four biological replicates. Three independent experiments were performed. One-way ANOVA analysis (Bewick *et al.* 2004) was used for significance testing. Knockout mutants were considered sensitive/slower growing (S) or resistant/faster growing (R) (Table 3) if there was a difference in percent growth of wild-type in the presence of the chemical at p<0.05.

### p38 MAPK assays

For analysis of MAPK profiles of the phosphatase mutants, conidia were used to inoculate VM liquid cultures at an initial concentration of 1 × 10^6^ conidia/ml as previously described (Jones and Borkovich 2010). Cultures were grown for 16 hr at 30° with shaking at 200 rpm and then treated with 0.8 M NaCl (for activation of p38 OS-2 MAPK) for 10 min. An untreated sample was used as an uninduced control (time zero). After treatment, the tissue was flash-frozen in liquid nitrogen and ground using 2-mm to 5-mm stainless steel beads (Qiagen) with the Qiagen Retsch TissueLyser system (Qiagen Retsch GmbH, Hannover, Germany). Depending on the amount of tissue, 300–700 μl extraction buffer (50 mM HEPES, pH 7.5; 2 mM EGTA; 2 mM EDTA; 1% SDS; 10% glycerol; 100 mM NaCl; 1 mM sodium orthovanadate; and 1 mM sodium fluoride) was added to the powdered fungal tissue, and the mixture was heated at 85° for 5 min. Afterwards, 10 μl of 100 mM PMSF and 1 μl of fungal protease inhibitor cocktail (Product #T8215; Sigma-Aldrich, St. Louis, MO) was added and the solution was centrifuged at 4000*g* for 15 min at 4°. The supernatant was collected and the protein concentration was determined using the BCA protein assay (Pierce Chemical, Rockford, IL). A volume of extract containing 30 μg protein was subjected to SDS-PAGE, followed by immunoblotting (Krystofova and Borkovich 2005). Commercial antibodies directed against mammalian or *S. cerevisiae* MAPKs were used to detect phospho-OS-2 (1:600 dilution; anti-phospho-p38 #9211; Cell Signaling Technology, Beverly, MA). Incubation with peroxidase-conjugated goat anti-rabbit IgG secondary antibody (Sigma Chemical, St. Louis, MO) and subsequent chemiluminescence detection was performed as previously described (Krystofova and Borkovich 2005).

## Results

### Protein phosphatase catalytic subunit genes in the *N. crassa* genome

We utilized the amino acid sequences of known protein phosphatase catalytic subunit genes from the Saccharomyces Genome Database (http://www.yeastgenome.org/) as queries during a reciprocal BLAST approach for identifying corresponding homologs in the Broad Institute *N. crassa* database (http://www.broadinstitute.org/annotation/genome/neurospora) (Table 1). We then performed additional BLAST searches and CDD domain analysis at NCBI to eliminate spurious small-molecule phosphatases. We identified 30 catalytic subunit genes that were classified as S/T (PPP, PPM, and Asp-based subfamilies) or PTPs (classical, dual-specificity, LMW-PTP, Cdc25-type, and SSU72 subfamilies). Two genes in the tyrosine phosphatase family (NCU01010 and NCU03333) appeared to be unique to filamentous fungi.

We compared protein phosphatases across different eukaryotic species (Table 2), including humans (Moorhead *et al.* 2007, 2009), a model plant, *A. thaliana* (Kerk *et al.* 2008; Moorhead *et al.* 2007), baker’s yeast, *S. cerevisiae* (Breitkreutz *et al.* 2010), and the filamentous fungi *A. nidulans* (Son and Osmani 2009), and *N. crassa*. There are many more S/T phosphatases in *A. thaliana* and humans than in the three fungi (Table 2). Comparing the fungi, *S. cerevisiae* has the greatest number (21 genes), followed by *A. nidulans* with 17 genes and *N. crassa* with 16 genes (Table 2). The observation of lower numbers of S/T phosphatase genes in *N. crassa* compared to *A. thaliana*, humans, and baker’s yeast is consistent with the fact that *N. crassa* has fewer S/T kinase genes (Park *et al.* 2011b).

**Table 2:** Serine/threonine and protein tyrosine phosphatase genes in *Homo sapiens*, *Arabidopsis thaliana*, *Saccharomyces cerevisiae*, *Neurospora crassa*, and *Aspergillus nidulans*

Serine/Threonine Protein Phosphatase Genes					
Family	*Homo sapiens* (Moorhead *et al.* 2007)	*Arabidopsis thaliana* (Moorhead *et al.* 2007; Kerk *et al.* 2008)	*Saccharomyces cerevisiae* (Moorhead *et al.* 2007; Breitkreutz *et al.* 2010)	*Neurospora crassa*	*Aspergillus nidulans* (Son and Osmani 2009)
PPP	13	26	11	8	8
PPM	18	76	7	5	6
Asp-based	13	23	3	3	3
Total	44	125	21	16	17
Protein Tyrosine Phosphatase Genes					
Classical	38	1	4	2	2
Dual-specificity	61	22	6	6	5
LMW-PTP	1	1	1	1	1
CDC25	3	—	2	2	1
SSU72	1	1	1	1	1
Y-phosphatase	—	—	—	2	1
Total	104	25	14	14	11

With regard to PTPs, the number of genes is similar in the three fungi but fewer than in humans and *A. thaliana* (Table 2). Despite the presence of tyrosine phosphatases, fungi do not possess recognizable tyrosine kinases (Borkovich *et al.* 2004; Kosti *et al.* 2010). The same is true for *A. thaliana* and several apicomplexan species whose genomes lack any true tyrosine kinases or receptor tyrosine kinases (Andreeva and Kutuzov 2008; Kerk *et al.* 2008; Moorhead *et al.* 2009). It is now believed that PTPs evolved before tyrosine kinases because of leaky phosphorylation of tyrosine residues by S/T kinases, thus providing a target for the tyrosine phosphatases (Moorhead *et al.* 2009).

As part of the *Neurospora* Genome Project, we attempted gene replacement of the 30 phosphatase genes (Colot *et al.* 2006). Transformants could not be recovered for ΔNCU00434 (*ptc-1*) and mutants for NCU05049 (*dsp-5*) were not available (Table 1). We were unable to purify four of the knockout mutants (ΔNCU03804; *cna-1*, ΔNCU00043; *ppp-1*, ΔNCU09300; *fcp-1* and ΔNCU06630; *pph-1*) to homokaryons. As mentioned, *cna-1* has been reported as an essential gene in *N. crassa* (Prokisch *et al.* 1997). The homolog for *pph-1* has been shown to be essential in *A. nidulans* (Son and Osmani 2009) and the gene was also shown to be essential for cell survival in *Neurospora* (Yang *et al.* 2004). Previous work showed that homologs of *ppp-1* and *fcp-1* are essential in both *A. nidulans* (Son and Osmani 2009) and *S. cerevisiae* (Archambault *et al.* 1997; Feng *et al.* 1991), and our observation the two *N. crassa* mutants could not be purified to homokaryons supports *ppp-1* and *fcp-1* as essential genes in *N. crassa*. Thus, for phenotypic analyses and characterization, we were able to analyze a total of 24 viable protein phosphatase mutants.

### Deletion of protein phosphatase genes leads to growth and developmental phenotypes in *N. crassa*

*N. crassa* is a heterothallic (self-sterile) fungus that spends most of its life cycle in the haploid state and grows vegetatively by apical extension of basal hyphae (Davis and Perkins 2002). The asexual phase of growth begins with germination of an asexual spore (conidium) that undergoes polarized growth to form hyphae. Hyphal fusion and branching give rise to the networked multicellular body of the organism, the mycelium. Different environmental stimuli, such as desiccation, heat, and/or nutrient deprivation, can stimulate the asexual sporulation pathway known as macroconidiation. This leads to the differentiation of aerial hyphae, which then bud from their tips, thus forming conidiophores and eventually giving rise to the free asexual spores, macroconidia or conidia (Springer 1993). Under nitrogen starvation, *N. crassa* enters into the sexual phase of development, inducing the formation of female reproductive structures known as protoperithecia (Raju and Leslie 1992). Chemotropic growth of a female hypha (trichogyne) toward a male cell (conidium) of opposite mating-type results in cell and nuclear fusion, followed by meiosis and enlargement of the protoperithecium into the fruiting body (perithecium). Perithecia contain the meiotic progeny known as ascospores that germinate to produce hyphae under appropriate environmental conditions (Raju and Leslie 1992).

**Figure 1:**
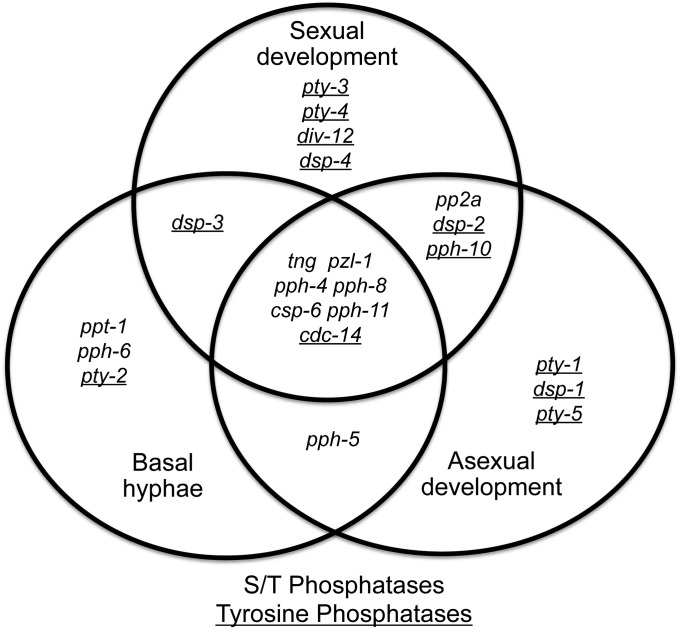
Venn diagram displaying distribution of protein phosphatase mutants with growth and developmental phenotypes. The 22 viable protein phosphatase mutants exhibiting defects in at least one major growth/developmental pathway are indicated by the names for deleted genes. The underlined gene names correspond to tyrosine phosphatases, whereas the remaining are serine/threonine phosphatases.

To characterize *N. crassa* phosphatase genes, we began with phenotypic analyses of the 24 viable mutants. In terms of extension of basal hyphae, nine mutants showed reduced growth and three displayed increased growth as compared to wild-type (Figure 1 and Table 1; detailed phenotypic data in Table S1). A total of 14 mutants exhibited defects in asexual development. Among these strains, only one mutant (Δ*pph-5*) possessed abnormalities in growth of basal hyphae and asexual development, but not in sexual development (Figure 1 and Table 1). The *pph-5* homolog in *S. cerevisiae* (*PTC5*) is also required for normal vegetative growth (Yoshikawa *et al.* 2011). Mutants lacking the genes *ppt-1*, *pph-6*, and *pty-2* displayed increased basal growth (compared to wild-type) as their only morphological phenotype. Interestingly, the *S. cerevisiae pty-2* homolog, *PTP1*, is a negative regulator of filamentation (Fasolo *et al.* 2011). The faster hyphal growth observed in the *N. crassa* Δ*pty-2* mutant suggests that *pty-2* and *PTP1* may have similar functions in *N. crassa* and *S. cerevisiae*.

Mutants lacking the DSP *dsp-1*, the CDC25 phosphatase *pty-1*, and the Y-phosphatase member *pty-5* exhibited phenotypes in asexual development as their only morphological defect. Deletion of the *dsp-1* homolog *PPS1* in *S. cerevisiae* does not produce any adverse effects on growth, but overexpression of *PPS1* results in growth arrest and aberrant DNA synthesis (Ernsting and Dixon 1997).

In our previous study of *N. crassa* kinases (Park *et al.* 2011b), we found that 32 out of 77 (42%) mutant strains exhibited defects in sexual development, with 30 out of 77 (39%) unable to produce ascospores (female-sterile; 94% of sexual phenotypes). In contrast, among the 24 phosphatase mutants, we found that 15 had a phenotype during sexual development (60%), with four strains (Δ*tng*, Δ*pp2A*, Δ*csp-6*, and Δ*dsp-2*; 16% of mutants) being female-sterile, accounting for 26% of the sexual phenotypes in the phosphatase mutants (Table 1, Figure 1, and Table S1). These results demonstrate that although a greater proportion of phosphatases than kinases influence the sexual cycle, kinases are more critical for production of ascospores and absolute female fertility in *N. crassa*.

The Δ*pp2A* mutant failed to produce protoperithecia and also had reduced aerial hyphae extension (Table 1, Figure 1, and Table S1). These phenotypes are similar to those of mutants lacking components of the two MAPK pathways in *N. crassa*: MIK-1/MEK-1/MAK-1 (cell fusion and cell wall integrity) and NRC-1/MEK-2/MAK-2 (cell fusion) (Kothe and Free 1998b; Li *et al.* 2005; Park *et al.* 2011b). However, the HAD class phosphatase knockout Δ*csp-6* was unique in that it had very few (and small) protoperithecia that were unable to mature into perithecia on fertilization with the opposite mating type.

Among the remaining strains with defects in sexual development, four mutants, Δ*pty-3*, Δ*div-12*, Δ*dsp-2*, and Δ*pph-10*, exhibited decreased numbers of protoperithecia and perithecia, as well as few or delayed shooting of ascospores (Table 1, Figure 1, and Table S1). The Δ*dsp-3* and Δ*dsp-4* each displayed abnormal and increased protoperithecia or perithecia formation and increased ascospore production (Table 1, Figure 1, and Table S1). It is interesting to note that all of these aforementioned genes are tyrosine phosphatases, suggesting that this phosphatase class is important for regulation of sexual development in *N. crassa*. Two more mutant strains (Δ*tng* and Δ*pph-11*) produced few protoperithecia and perithecia, and whereas one ejected no ascospores (Δ*tng*), the other produced very few (Δ*pph-11*). Another three mutants (Δ*pph-4*, Δ*cdc-14*, and Δ*pph-8*) possessed defects in the timing or in the number of ascospores produced. The *pph-4* mutant developed abnormal/small protoperithecia but normal-appearing perithecia, whereas Δ*pph-8* was precocious in protoperithecia formation, leading to perithecia that were embedded in the agar surface. A null mutation in the well-characterized phosphatase *CDC14* (involved in mitotic exit and meiosis I spindle disassembly) is lethal in yeast (Taylor *et al.* 1997), whereas the *N. crassa* Δ*cdc-14* mutant is viable (but with defects in all three growth/developmental pathways). Deletion of *cdc-14* in *A. nidulans* did not result in any obvious growth defects (Son and Osmani 2009).

Most of the 15 mutants that had phenotypes in sexual development also exhibited defects in basal hyphae extension and asexual differentiation. However, Δ*pty-1*, Δ*dsp-1*, and Δ*pty-5* demonstrated phenotypes only during asexual development and thus seem to be specific for aspects of conidiation in *N. crassa* (Figure 1, Table 1, and Table S1). Overall, our results show that 22 out of 24 mutants (91%) displayed a defect in at least one of three growth/developmental pathways analyzed in this study (Figure 1 and Table 1), with seven of the 24 mutants (29%) possessing phenotypes in all three stages. As a comparison, among the previously studied S/T protein kinase knockouts in *N. crassa* (Park *et al.* 2011b), 57% of the mutants possessed a defect in at least one of the growth/developmental stages, whereas 45% had overlapping defects in all three. This suggests that similar to kinases, phosphatases are also important regulators of growth and development in *N. crassa*.

### Chemical sensitivity assays reveal additional phenotypes for protein phosphatase mutants

Various chemical and environmental stresses have been known to influence growth and developmental outcomes in eukaryotic cells. To gain a better understanding of the functions of the different protein phosphatases in *N. crassa*, we subjected the phosphatase mutants to a panel of chemical treatments (see *Materials and Methods*) and compared their relative sensitivity to each chemical to that of wild-type (Table 3; detailed results in Table S1). Strains with linear growth rates less than 50% of wild-type on minimal medium were excluded from this assay to avoid any bias attributable to their slow growth.

**Table 3:** Mutants with chemical sensitivity phenotypes

NCU	FGSC	Deleted Gene	Sodium Chloride	Sorbitol	Cytochalasin A	Benomyl	Tert-Butyl Hydroperoxide	Menadione	FK506	Fludioxonil	Yeast Extract
06563	11546	*pp2a*	S	S		R	S	S		R	
07489	11548	*pzl-1*	R	R		R	S	S	R		S
08301	12454	*pph-4*			R		S	S			S
01433	15790	*ppt-1*					R				
00958	19378	*pph-7*								S	
01767	12451	*pph-5*					R				
03495	16430	*pph-6*					S		S		
08380	20306	*csp-6*	S			R	S		R	S	
02257	16060	*pty-2*					R		R		
05364	12444	*pty-3*		S							
03246	13311	*cdc-14*		R		R			R		
03426	16425	*dsp-1*					R				
06252	14464	*dsp-2*		S			S				
06330	15781	*dsp-3*									
08158	19644	*dsp-4*									
09841	18801	*pty-4*						R	R		
02496	16654	*div-12*								R	
06966	14056	*pty-1*									
03114	16337	*pph-10*			R						
01010	16679	*pty-5*									
0333	17653	*pty-6*							R		

One-way ANOVA analysis was performed to determine significance. These results reflect strains displaying chemical sensitivity phenotypes at p<0.05. Radial colony growth was measured and percentage growth was calculated as growth with chemical *vs.* growth without chemical. See *Materials and Methods* for details.

Mutants were classified as sensitive (S) or resistant (R) relative to the growth of wild-type.

We analyzed the relative sensitivities of the phosphatase mutants to the reactive oxygen species (ROS) generating chemical menadione (Loor *et al.* 2010), whereas peroxide stress was introduced by exposure to *t*-BuOOH (Kim *et al.* 2012). Similar to our previous study of kinases (Park *et al.* 2011b), treatment with *t*-BuOOH yielded the greatest number of phenotypes, with a total of 10 strains displaying sensitivity or resistance to peroxide treatment. Three mutants (Δ*pp2A*, Δ*pzl-1*, and Δ*pph-4*) showed increased sensitivity to both *t*-BuOOH as well as menadione (Table 3), whereas Δ*pph-6*, Δ*csp-6*, and Δ*dsp-2* were exclusively sensitive to peroxide. The tyrosine phosphatase mutant Δ*pty-4* was resistant to menadione treatment, whereas Δ*ppt-1*, Δ*pph-5*, Δ*pty-2*, and Δ*dsp-1* were resistant to *t*-BuOOH. It is thus of particular interest to understand how these phosphatases might be regulating cellular responses to oxidative stress. Conidiation is known to be influenced by ROS in *N. crassa* (Hansberg *et al.* 1993; Toledo *et al.* 1994). In the case of the Δ*csp-6*, we have shown that this mutant is defective in conidial separation (Figure 4). The finding that it is also sensitive to peroxide stress reinforces the notion that *csp-6* is an important component of the conidiation pathway in *N. crassa*.

We used sodium chloride and sorbitol to induce salt/osmotic stress in the *N. crassa* phosphatase mutants. A total of six mutants exhibited phenotypes in these assays. The Δ*pp2A* mutant was sensitive to both sorbitol and sodium chloride, suggesting it has important roles in osmotic stress resistance. The *S. cerevisiae* homolog of *pzl-1*, PPZ1, has been characterized as an important (negative) regulator of salt stress, halo tolerance, and pH homeostasis (Posas *et al.* 1995; Yenush *et al.* 2002). In our assays, we found that the Δ*pzl-1* mutant was resistant to both sorbitol and sodium chloride, providing evidence for similar functions for *pzl-1* in *N. crassa* as observed in baker’s yeast. *S. cerevisiae* Psr1p and Psr2p are most similar to *N. crassa* PPH-11 and CSP-6, respectively (Siniossoglou *et al.* 2000) (Table 1). The slow growth rate of the *N. crassa* Δ*pph-11* mutant disqualified this strain for chemical sensitivity screening, but we observed that the Δ*csp-6* mutant was sensitive to sodium chloride (Table 3). The observation that Δ*psr1* and Δ*psr2* single mutants are normal but that loss of both genes leads to sensitivity to salt stress in *S. cerevisiae* (Siniossoglou *et al.* 2000) illustrates the difference in genetic wiring between *S. cerevisiae* and *N. crassa*.

To decipher possible functions for protein phosphatases in cytoskeletal maintenance, we treated the mutants with cytochalasin A, which prevents polymerization and elongation of actin filaments (Cooper 1987) and benomyl, which binds to microtubules, thus inhibiting mitosis, meiosis, and cellular transport (Willhite 1983). Interestingly, the only phenotype observed using these chemicals was increased resistance. Two mutants, Δ*pph-4* and Δ*pph-10*, were resistant to cytochalasin A, whereas four (Δ*pp2A*, Δ*pzl-1*, Δ*cdc-14*, and Δ*csp*-*6*) showed enhanced growth as compared to wild-type with benomyl treatment (Table 3). It is possible that these missing phosphatases play important antagonistic roles in mitotic exit or in cell proliferation, perhaps through dephosphorylation of a mitotic/cell proliferation kinase. For example, *cdc-14* is known to be an important regulator of the cell cycle and mitosis in fungi and deletion of the gene in *N. crassa* imparts resistance to benomyl. Deletion of *cdc-14* leads to increased activity of *cdk-1*, which is known to promote cell proliferation and survival (in mammalian cells) via phosphorylation of the transcription factor FOXO1 (Liu *et al.* 2008; Stegmeier and Amon 2004). Resistance to benomyl in Δ*cdc-14* might be imparted via a similar mechanism, which in effect counteracts the inhibitory effects of the chemical.

FK506 is a macrolide lactone (Dumont *et al.* 1990) that binds the immunophilin FKBP12 (FK506 binding protein), inhibiting the S/T phosphatase calcineurin in the calcium-signaling pathway (Prokisch *et al.* 1997). Assays with this immunosuppressant drug revealed that six mutants (Δ*pzl-1*, Δ*csp-6*, Δ*pty-2*, Δ*cdc-14*, Δ*pty-4*, and Δ*pty-6*) were resistant, whereas one (Δ*pph-6*) was sensitive to FK506 (Table 3). Resistance to FK506 was the only phenotype for the Δ*pty-6* mutant.

Calcineurin A subunit mutants have been shown to have phosphatase activity with increased resistance to FKBP12-FK506 in mammalian cells (Kawamura and Su 1995). A similar effect is also observed in case of TOR pathway mutants in yeast (*TOR1* and *TOR2*) that display resistance to a different macrolide, sirolimus, also known as rapamycin (Dumont *et al.* 1990). The TOR signaling pathway has also been implicated in regulation of microtubule structure/function and acts antagonistically to the calcineurin-signaling network (Choi *et al.* 2000; Mulet *et al.* 2006). Of the strains that were resistant to FK506, three mutants (Δ*pzl-1*, Δ*csp-6*, and Δ*cdc-14*) also displayed resistance toward benomyl. It is thus tempting to speculate that these three phosphatase knockout mutants with a common resistance to benomyl and FK506 might have overlapping roles in the TOR pathway and calcineurin function in *N. crassa*.

Fludioxonil is a phenylpyrrole class fungicide (Ochiai *et al.* 2001) that stimulates the OS-2 MAPK pathway, leading to increased glycerol production and cell death in *N. crassa* (Zhang *et al.* 2002). The OS MAPK module mutants (*os*-4/*os*-5/*os*-2) are resistant to fludioxonil but sensitive to sodium chloride and sorbitol (Park *et al.* 2011b; Zhang *et al.* 2002). We found that two mutants, Δ*csp-6* and Δ*pph-7*, are sensitive to fludioxonil, whereas the PP2A class phosphatase mutant Δ*pp2A* and CDC25 phosphatase mutant Δ*div-12* were resistant. The resistance phenotype of the latter group suggests that these gene products might have important roles in the OS-2 MAPK signaling pathway. Incidentally, fludioxonil sensitivity was the only phenotype observed for the Δ*pph-7* mutant in this study.

We also analyzed the relative growth of the phosphatase mutants on medium supplemented with 2% yeast extract, which is rich in amino acids, peptides, and vitamins. Only two phosphatase mutants, Δ*pzl-1* and Δ*pph-4*, exhibited a significant difference in growth relative to wild-type on 2% yeast extract. Both of these strains grow less well than wild-type, suggesting that nutrient sensing and/or utilization abilities are compromised in the mutants.

The 24 viable phosphatase mutants were also cultured on VM with Avicel (2%) substituted for sucrose as an alternate carbon source. We found that two phosphatase mutants (Δ*pph-4* and Δ*dsp-2*) were better able to utilize Avicel than wild-type, consistent with the corresponding genes acting as negative regulators of cellulose utilization (Table 4). It is interesting to note that both of these mutants display a common sensitivity to *t*-BuOOH, and that Δ*pph-4* is also sensitive to menadione. It will be of interest in future studies to determine whether sensitivity to oxidative stress could prove beneficial in upregulating carbon metabolism genes in fungi and how these phosphatases could assist in the process.

**Table 4:** Mutants with altered growth on 2% Avicel

Strains	Sucrose (mm/day)*^a^*	Avicel (mm/d)*^b^*	% Growth*^c^*	SD*^d^*
Wild-type (*mat a*)	33.6	18.2	54	0.106
Δ*pph-4*	18	14	78	0.114
Δ*dsp-2*	29.5	22.83	77	0.100

a Radial growth of strains on minimal medium containing sucrose.

b Radial growth of strains on minimal medium containing 2% Avicel.

c % Growth = (radial growth on Avicel)/(radial growth on sucrose) × 100.

d SD for three replicates.

Through our morphological testing, we determined that two of the 24 viable mutants had no obvious growth defects (Δ*pty-6* and Δ*pph-7*). However, phenotypes were revealed for these two mutants through the chemical sensitivity assays, resulting in at least one phenotype for every phosphatase mutant analyzed. This supports the advantage of chemical testing for identifying defects for mutants that do not display growth or developmental phenotypes. Knockout strains for *pzl-1* and *pp2a* possessed the greatest number of chemical sensitivity phenotypes and 17 of 21 tested strains exhibited at least one chemical sensitivity phenotype. Also, taking into account mutants that possessed either significant sensitivity or resistance to more than one chemical, we observed a total of 42 chemical sensitivity phenotypes for a set of 21 phosphatase mutants.

### The phospho-p38 MAPK level is elevated in a number of protein phosphatase mutants

MAPKs are a class of S/T kinases present in all eukaryotic cells. As a group, they are responsible for a wide variety of cellular responses toward stress and environmental stimuli and also regulate gene expression, metabolism, mitosis, apoptosis, cellular motility, and differentiation (Cargnello and Roux 2011; Gehart *et al.* 2010; Kukkonen-Macchi *et al.* 2011; Nelson and Fry 2001; Paliwal *et al.* 2007). MAPKs are highly conserved throughout evolution and also are one of the most widely studied groups of proteins for investigation of physiological responses (Widmann *et al.* 1999). The p38 MAPK homologs in *S. cerevisiae* (Hog1p) and *N. crassa* (OS-2) are involved in cellular responses to hyperosmolarity as well as oxidative stress (Banno *et al.* 2007; Lamb *et al.* 2012; Staleva *et al.* 2004). Previous studies have shown that loss of any of the three genes in the MAPK module (*os-4*, *os-5*, and *os-2*) in *N. crassa* does not appreciably affect basal hyphal growth but rather leads to fragile conidia, increased sensitivity to hyperosmotic conditions, resistance to the fungicide fludioxonil, and female sterility (Jones *et al.* 2007; Zhang *et al.* 2002).

**Figure 2:**
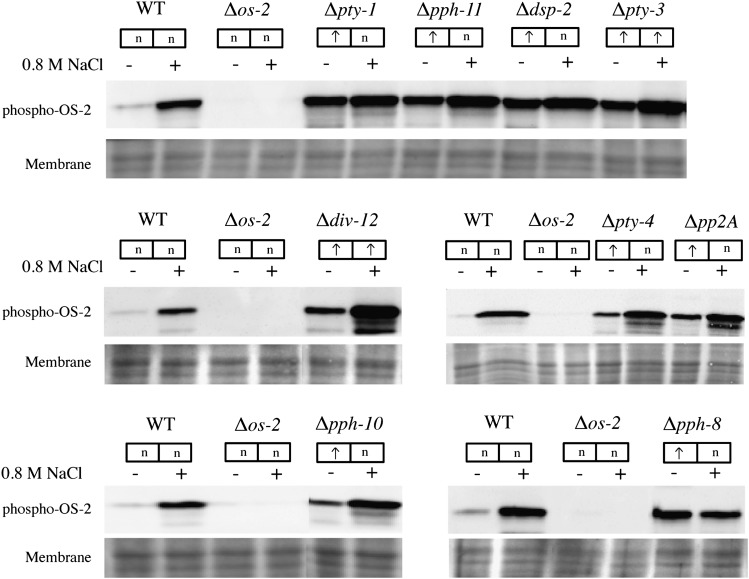
Analysis of p38 MAPK phosphorylation. Conidia were used to inoculate shaken liquid cultures that were grown for 16 hr at 30°. Cultures were left untreated or brought to 0.8 M NaCl for 10 min to stimulate OS-2 phosphorylation. Phospho-OS-2 levels were analyzed by immunoblotting with a specific antiserum (top panels). A portion of the membrane was excised and stained using amido black to use as a loading control (bottom panels). The experiment was repeated at least three times and a representative blot is shown. The letter “n” signifies that the levels of phospho-OS-2 were similar to wild-type. The arrow signifies that the levels of phospho-OS-2 were elevated as compared to wild-type.

To identify protein phosphatases that may act on the OS MAPK cascade, we analyzed the phosphorylation status of OS-2 in the 24 viable phosphatase knockouts using 0.8 M NaCl for inducing osmotic stress in 16-hr liquid cultures (see *Materials and Methods*). For detecting the phosphorylated form of OS-2 in the protein samples from the cellular extracts, we used commercially available peptide antibodies raised against the mammalian MAPK phospho-p38. Similar to previous studies (Jones *et al.* 2007), this antibody was found to cross-react with a *N. crassa* phosphoprotein of ~41 kDa, which is near the predicted size of OS-2 (Figure 2). We found that nine mutants had elevated basal levels of phospho-OS-2: Δ*pty-1*, Δ*pph-11*, Δ*dsp-2*, Δ*pty-3*, Δ*div-12*, Δ*pty-4*, Δ*pp2A*, Δ*pph-10*, and Δ*pph-8* (Figure 2 and Table 1). However, only Δ*div*-12 and Δ*pty-3* exhibited significantly higher levels of phospho-OS-2 than wild-type after induction using sodium chloride (Figure 2). As mentioned, Δ*div-12* also displays resistance to fludioxonil (Table 3), a phenotype similar to that of mutants lacking *os-2*, *os-4*, or *os-5*. Hence, DIV-12 (a CDC25-type PTP) may play a role in dephosphorylation of one or more of the component kinases of the OS pathway in *N. crassa*. In budding yeast, Mih1p (a DIV-12 homolog) is involved in dephosphorylation of CDC28 (Russell *et al.* 1989; Sia *et al.* 1996) and a role in Hog1p dephosphorylation has also been suggested (Clotet *et al.* 2006). The other CDC25-type phosphatase in *N. crassa*, PTY-1, also seems to have functions in the OS-2 pathway, because deletion of *pty-1* leads to an increase in the basal levels of phospho-OS-2. We also found that Δ*pph-10* showed elevated basal phospho-OS-2. The yeast homolog SSU72 phosphatase is primarily involved in transcription termination via removal of phospho-Ser7 marks from the RNA Pol II CTD (Zhang *et al.* 2012). Our MAPK assays also implicate the SSU72 class of phosphatases in regulation of OS-2 phosphorylation, although such a function might also be imparted via regulation of the transcriptional machinery.

Deletion of the PTPs *pty-3* and *dsp-2* leads to increased basal levels of phospho-OS-2 in comparison to wild-type (Figure 2). The levels of phospho-OS-2 in the Δ*pty-3* strain are also elevated on induction using sodium chloride (as compared to wild-type). The yeast *pty-3* homolog PTP3 is involved in dephosphorylation of both Hog1p and Slt2p in the cell wall integrity pathway (Hahn and Thiele 2002; Wurgler-Murphy *et al.* 1997). In contrast, Msg5p, the yeast homolog of DSP-2, is implicated in dephosphorylation of Slt2p and Fus3p in the pheromone-sensing pathway (Andersson *et al.* 2004; Flandez *et al.* 2004; Marin *et al.* 2009). Our results suggest that DSP-2 is also required for inactivation of the OS-2 pathway, and further study of the Erk class MAPKs MAK-1 and MAK-2 in *N. crassa* may uncover similar functions for this phosphatase as seen in yeast. It is also possible that there is a high degree of crosstalk between the different MAPK cascades, leading to an overlap of function.

Our study of the LMW-PTP *pty-4* is especially unique because, so far, a cellular role has not been demonstrated in *S. cerevisiae* (Ostanin *et al.* 1995). The *N. crassa* Δ*pty-4* mutant exhibits decreased production of protoperithecia and resistance toward menadione and FK-506 (Table 1, Table 3, and Table S1). This suggests that PTY-4 negatively regulates pathways involved in activation of oxidative stress responses. Deletion of *pty-4* leads to an increased basal level of phospho-OS-2 as compared to wild-type (Figure 2). These results suggest that PTY-4 may regulate sexual development and ROS sensitivity via OS-2 phosphorylation in *N. crassa*.

Among the PP2A class of S/T phosphatases, Δ*pp2A* was the only mutant exhibiting elevated basal phospho-OS-2 levels (Figure 2). The finding that the *pp2A* mutant was sensitive to sodium chloride and sorbitol but resistant to fludioxonil treatment also supports a role as a major phosphatase in the OS-2 pathway (Table 3). The PP2C class phosphatase mutant Δ*pph-8* and the HAD class mutant Δ*pph-11* also exhibited elevated levels of basal phospho-OS-2 (Figure 2). The *S. cerevisiae* homolog of *pp2A* is *PPG1* and that for *pph-8* is *PTC2*; the *PPG1* and *PTC2* gene products are required for glycogen accumulation and dephosphorylation of Hog1p, respectively (Posas *et al.* 1993; Young *et al.* 2002). These results suggest that similar to their homologs in yeast, these phosphatases are bona fide regulators of OS-2 dephosphorylation and have important roles in the MAPK signaling cascade.

Taken together, our assays revealed that 9 out of 24 viable mutants exhibit altered p38 MAPK phosphorylation. Information for a number of these phosphatases in *S. cerevisiae* indicates that there is considerable crosstalk and/or overlap in function for some of these phosphatases with other MAPK cascades, such as the cell wall integrity pathway (Andersson *et al.* 2004; Gonzalez *et al.* 2006; Hahn and Thiele 2002; Isoda *et al.* 2009). Our results suggest that a similar commonality in function may also exist in *N. crassa*.

### Deletion of the PP2C class protein phosphatase gene *pph-8* leads to unregulated protoperithecial development in *N. crassa*

**Figure 3:**
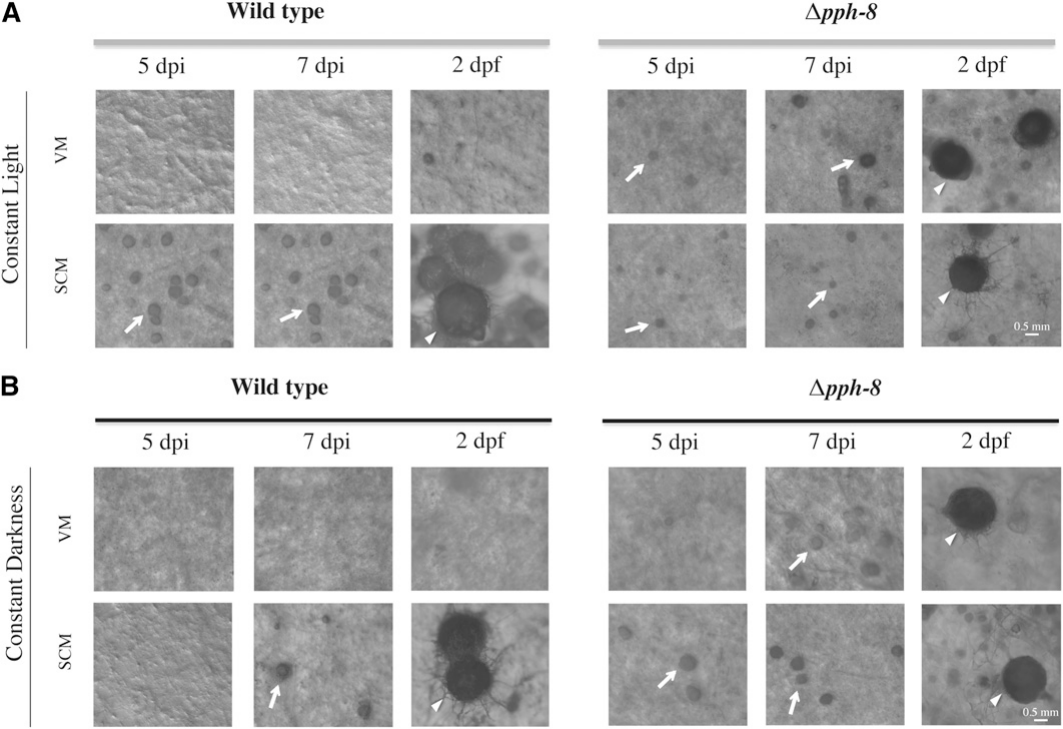
The Δ*pph-8* mutant displays unregulated protoperithecial development on minimal medium. Wild-type and Δ*pph-8* strains were cultured on VM and SCM plates under constant light (A) or constant darkness (B) and photographed at 5 d postinoculation (dpi), 7 dpi, and 2 d postfertilization (dpf) with opposite mating-type conidia. The white arrows point to protoperithecia, whereas the white arrowheads indicate mature perithecia. Scale bar = 0.5 mm.

In *N. crassa*, the formation of female sexual structures (protoperithecia) is stimulated by growth on SCM containing low nitrogen (Sommer *et al.* 1987). While performing phenotypic analysis of the phosphatase mutants, we observed that a PP2C class phosphatase knockout mutant (Δ*pph-8*) displayed inappropriate protoperithecial formation on VM medium containing high nitrogen (Figure 3). To further assess this unregulated protoperithecial formation, Δ*pph-8* was cultured on VM and SCM agar plates in constant light or constant darkness for 5 d to 7 d and then fertilized with an opposite mating type wild-type strain. At 5 d under constant light conditions, the Δ*pph-8* mutant displayed small protoperithecia that were embedded under the agar surface of both media. At 7 d, Δ*pph-8* protoperithecia differentiated on VM had enlarged to the same size seen in the wild-type strain at 7 d on SCM medium, whereas Δ*pph-8* protoperithecia produced on SCM were smaller than wild-type (Figure 3A). After fertilization, the Δ*pph-8* mutant was able to form mature perithecia (Figure 3A) and shoot ascospores 10 days after fertilization (data not shown). Previous studies have shown that blue light is necessary for photo-induction of protoperithecial development (Innocenti *et al.* 1983). When cultured in constant darkness, wild-type produced protoperithecia after 7 d on SCM (but not VM), a delay of 2 d relative to constant light conditions (Figure 3B). The Δ*pph-8* mutant exhibited no delay in protoperithecial development on either VM or SCM in constant darkness (Figure 3B). Interestingly, Δ*pph-8* protoperithecia formed in constant darkness were slightly larger than those formed in light (Figure 3). Perithecia were produced in wild-type on SCM and the Δ*pph-8* mutant on both SCM and VM medium in constant darkness (Figure 3). When formed, perithecia from wild-type and the Δ*pph-8* mutant produced ascospores by 10 d after fertilization (data not shown). However, in contrast to wild-type, Δ*pph-8* ascospore progeny from VM cultures (light or dark conditions) did not germinate (data not shown).

The results presented suggest that loss of *pph-8* significantly affects nitrogen sensing and the sexual development pathway in *N. crassa*. This mutant also exhibited multiple defects in hyphal growth and asexual sporulation (Table 1 and Table 3). PPH-8 shares a high degree of homology to Ptc2p in *S. cerevisiae* (Young *et al.* 2002). Ptc2p dephosphorylates Hog1p as well as Cdc28p, and is also implicated in functioning with proteins such as RAD53 to regulate DNA damage checkpoint pathways (Cheng *et al.* 1999; Marsolier *et al.* 2000; Young *et al.* 2002). As seen from the MAPK assays, Δ*pph-8* has a high basal level of phospho-OS-2, and levels after treatment with sodium chloride are similar to those of treated wild-type. This suggests that PPH-8 is involved in dephosphorylation of the OS-2 MAPK in *N. crassa* (Figure 2). Other studies have shown that the *os-4*/*os-5*/*os-2* mutants are unable to produce protoperithecia (Jones *et al.* 2007), a phenotype in opposition to that observed for Δ*pph-8*. Hence, it is plausible that the PPH-8 phosphatase regulates protoperithecial development via modulation of the OS-2 MAPK pathway.

### The protein phosphatase mutant Δ*csp-6* displays a conidial separation defect most similar to Δ*csp-1* mutants

Two conidial separation mutants, Δ*csp-1* and Δ*csp-2*, form major constriction chains with double crosswalls in developing conidiophores but no free macroconidia (Selitrennikoff *et al.* 1974). Genetic and molecular studies that have implicated a number of genes in the macroconidiation pathway place *csp-1* and *csp-2* downstream of other genes, including *acon-2* and *fl* (Bailey-Shrode and Ebbole 2004; Springer and Yanofsky 1989). The *csp-1* gene encodes a light-inducible zinc finger transcription factor, and deletion of *csp-1* leads to shortening of the period length for the circadian clock by approximately 1 hr (Lambreghts *et al.* 2009; Schneider *et al.* 2009). Recent evidence showed that CSP-1 is a transcription repressor, with its function and abundance coupled to the circadian activity of the white collar complex (WCC), thus constituting an important output for the clock (Sancar *et al.* 2011; Smith *et al.* 2010). CSP-1 is primarily involved in ergosterol biosynthesis, modulating the lipid composition of membranes (Sancar *et al.* 2011; Smith *et al.* 2010). In contrast to *csp-1*, deletion of the grainy head transcription factor gene *csp-2* lengthens the clock period by 1.5 hr in *N. crassa* (Brody *et al.* 2010; Pare *et al.* 2012). Among its functions, CSP-2 influences expression of genes involved in construction and remodeling of the cell wall (Pare *et al.* 2012).

**Figure 4:**
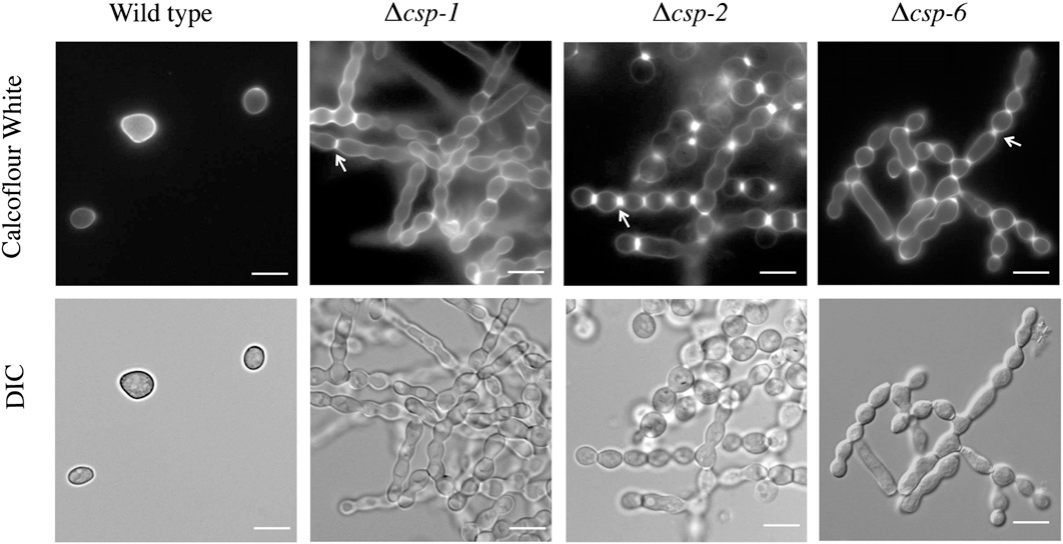
The Δ*csp-6* displays a conidiation separation defect most similar to Δ*csp-1* strains. Wild-type, Δ*csp-1*, Δ*csp-2*, and Δ*csp-6* strains were cultured on VM medium for 7 d under constant light and conidia were stained with calcofluor white to visualize developing crosswalls in the conidial chains. The arrow points to the conjoined conidia, indicating the separation defect. Scale bar size = 10 μ.

Microscopic observation of conidia revealed that Δ*csp-6*, lacking a HAD class S/T phosphatase, appeared to possess a conidial separation defect reminiscent of Δ*csp-1* and Δ*csp-2* mutants. Similar to the Δ*csp-1* and Δ*csp-2* strains, when slant cultures of Δ*csp-6* are agitated, no free conidia are released. In addition to a conidial separation defect, the Δ*csp-6* mutant exhibited reduced basal hyphal growth and produced few, small protoperithecia that did not develop into mature perithecia after fertilization during sexual development (Table 1 and Table S1). This contrasts with knockout mutants lacking *csp-1* or *csp-2*, which have reduced hyphal growth but do not possess defects in female sexual development (Broad database). To more accurately compare and contrast the conidial separation defects of Δ*csp-1*, Δ*csp-2*, and Δ*csp-6* strains, we used the fluorescent stain calcofluor white to visualize the cell wall (Fig. 4). On staining with calcofluor white, we found that the Δ*csp-2* mutant is able to form numerous double-doublets at interconidial junctions (Fig. 4), consistent with results from a previous study (Springer and Yanofsky 1989). This suggests that the Δ*csp-2* mutant is blocked at the double-doublet stage before connective formation takes place. Consistent with previous results, we also observed that the Δ*csp-1* mutant displays fewer double-doublets and sometimes does not form septa between macroconidial compartments (Figure 4) (Springer and Yanofsky 1989). In the case of the Δ*csp-6* mutant, we observed double-doublets (Fig. 4), but such structures were not as extensive as in Δ*csp-2* strains. In addition, the *csp-6* mutant sometimes lacked septa between macroconidial compartments in conidiophores (Figure 4). Hence, the conidial separation defect of Δ*csp-6* is more similar to Δ*csp-1* than to Δ*csp-2*. This conclusion supports CSP-1 and CSP-6 acting in the same pathway to regulate growth and conidiation, perhaps through dephosphorylation of phosphorylated CSP-1 transcription factor (or a regulated target) by the CSP-6 protein phosphatase.

## Discussion

In this study, we examined the role of protein phosphatases in growth and development and regulation of p38 MAPK dephosphorylation in the filamentous fungus, *N. crassa*. We have identified 30 protein phosphatase genes in the *N. crassa* genome and found that these genes are highly conserved among humans, plants, and other fungi. In particular, *N. crassa* phosphatases are in number similar to *A. nidulans*. Two phosphatases (*pty-5* and *pty-6*) showed little or no homology to genes in yeast, animals, or plants, whereas similar genes are present in *A. nidulans*, suggesting that these are specific for filamentous fungi. Our results demonstrated that Δ*pty-5* mutants possess defects in conidiation, whereas strains lacking *pty-6* are resistant to fludioxonil. Conidiation is observed in many filamentous fungi, but not baker’s yeast. Likewise, in contrast to many filamentous fungal species, *S. cerevisiae* is naturally resistant to fludioxonil, apparently lacking the cellular target of this fungicide (Motoyama *et al.* 2005; Zhang *et al.* 2002). Future studies will shed light on the cellular pathways impacted by these two tyrosine phosphatases in filamentous fungi.

A majority of protein phosphatase knockouts (91%) exhibited defects in basal growth, asexual development, or sexual development. We found that three mutants (Δ*pty-2*, Δ*pph-6*, and Δ*ppt-1*) actually displayed increased basal growth rates compared to wild-type. This is in contrast to our previous study with kinase mutants (Park *et al.* 2011b), in which all mutants with a basal hyphae growth defect exhibited reduced growth. Because, in general, phosphatases impart their roles by dephosphorylation of their targets, it is likely that phosphatase mutants with increased growth may experience constitutive phosphorylation of targets, leading to unregulated cell proliferation. Interestingly, the Δ*pty-2* and Δ*ppt-1* mutants were resistant to *t*-BuOOH, perhaps suggestive of a link between increased growth and oxidative stress resistance.

Morphological analyses of two development pathways in *N. crassa* showed that certain phosphatases are specific for sexual or asexual development (Figure 1). In particular, four tyrosine phosphatases (*pty-3*, *pty-4*, *div-12*, and *dsp-4*) are restricted to sexual development, whereas another three tyrosine phosphatases (*pty-1*, *dsp-1*, and *pty-5*) are only involved in asexual differentiation. In contrast, S/T phosphatases seem to have broader roles in fungal development (Figure 1). Deletion of various S/T kinases in *N. crassa* led to a high proportion (40%) of female-sterile strains (Park *et al.* 2011b). In contrast, only four phosphatase mutants (Δ*tng*, Δ*pp2A*, Δ*csp-6*, and Δ*dsp-2*) were female-sterile, representing 16% of the viable phosphatase mutants. This may reflect the antagonistic roles of protein phosphatases and kinases, with constitutive phosphorylation of targets in phosphatase mutants less likely to result in female sterility. Opposing functions for kinases and phosphatases are also manifested by the chemical sensitivity phenotypes. Treatment of S/T kinase mutants with *t*-BuOOH only revealed strains with increased sensitivity (Park *et al.* 2011b), whereas 40% of the affected phosphatase mutants displayed a resistant phenotype.

Two of the analyzed protein phosphatase mutants (Δ*pph-7* and Δ*pty-6*) did not have obvious growth or developmental phenotypes. As in our previous study of kinases, chemical sensitivity assays proved to be an effective tool in assigning a function for such mutants lacking a morphological phenotype. Deletion of *pty-6* resulted in increased resistance to the calcineurin inhibitor FK506, whereas the absence of *pph-7* rendered the strain sensitive to fludioxonil (which stimulates the OS-2 pathway) (Table 3). In *S. cerevisiae*, deletion of the *pty-6* homolog SIW14 leads to cytoskeletal abnormalities and defective endocytosis (Care *et al.* 2004). Hence, it is possible that *pty-6* and its related phosphatases might regulate cytoskeletal organization in concert with the calcineurin-mediated signaling pathways in *N. crassa*. In the case of *pph-7*, an intron in the mRNA of the *S. cerevisiae* homolog *PTC7* is alternatively spliced, producing two protein isoforms (Juneau *et al.* 2009). The protein derived from the spliced mRNA is localized to the mitochondrion, whereas that produced from the unspliced mRNA is found on the nuclear envelope. The mitochondrial protein is modified in a carbon source–dependent fashion, whereas mutants lacking the version on the nuclear envelope are more sensitive to latrunculin (a chemical that disrupts actin filaments) than wild-type (Juneau *et al.* 2009). In contrast to its closest yeast homolog, *N. crassa pty-6* lacks an intron in the ORF and biochemical studies have localized the protein to the mitochondrion (Keeping *et al.* 2011). Furthermore, we did not observe altered sensitivity of the Δ*pty-6* mutant to cytochalasin A, but instead to fludioxonil, which has been shown to activate the OS-2 MAPK pathway, leading to glycerol production. No proteins involved in fludioxonil sensitivity have been localized to the mitochondrion. Our findings support a scenario in which loss of *pty-6* leads to elevated production of glycerol in *N. crassa*. This likely occurs at a point downstream of the OS-2 MAPK, because we observed that Δ*pty-6* mutants possessed normal basal and induced levels of phospho-OS-2. Concomitant loss of *pty-6* and inappropriate activation of the OS-2 MAPK by fludioxonil would render the mutant more sensitive than wild-type.

A number of studies in *N. crassa* investigating utilization of cellulose as an alternate carbon source have shown that there is an upregulation of lignocellulolytic enzymes when *N. crassa* is switched from sucrose to cellulose (Sun and Glass 2011; Tian *et al.* 2009; Wu *et al.* 2013; Znameroski *et al.* 2012). Previous work has identified the zinc finger transcription factor CRE-1 as a carbon catabolite repressor, whereby deletion of *cre-1* leads to increased expression of cellulolytic genes when *N. crassa* is grown on the microcrystalline cellulose source, Avicel (Sun and Glass 2011). We have identified two protein phosphatase mutants, Δ*pph-4* and Δ*dsp-2*, that display increased growth on Avicel, consistent with roles as negative regulators of cellulose utilization. Therefore, it is possible that these phosphatases may operate in the same pathway or play parallel roles with CRE-1 in regulating the transcriptional machinery or downstream events to influence cellulolytic activity in *N. crassa*.

The phosphatases *pp2A* (NCU06563) and *pzl-1* (NCU07489) belong to the PP2A class of S/T phosphatases, a highly conserved family of proteins with several important functions in cellular signaling, from mammals to fungi (Du *et al.* 2013; Erental *et al.* 2007; Seshacharyulu *et al.* 2013). It is therefore not surprising that deletion of these genes led to several defects in growth and development and also yielded the highest number of chemical sensitivity phenotypes (six for Δ*pp2A* and seven for Δ*pzl-1*). The Δ*pp2A* mutant was sensitive to osmotic stresses, peroxide stress, and ROS, as well as displaying resistance to fludioxonil and benomyl (Table 3). All of these chemical sensitivity phenotypes were observed with high significance and very low p-values, and the p38 MAPK assays further reaffirm the authenticity of the responses for this mutant to the respective chemicals (Table S1). From the p38 MAPK assays, we found that Δ*pp2A* displays elevated levels of basal phospho-OS-2 (Figure 2), and its function in the OS-2 pathway is also reflected by the resistance of the mutant to fludioxonil, similar to the *os* mutants (Park *et al.* 2011b; Zhang *et al.* 2002). It remains to be investigated whether this phosphatase plays a direct role in dephosphorylation of the terminal MAPK OS-2 or is acting on the upstream MAPKKK (OS-4) or MAPKK (OS-5). This is additionally interesting when considering that OS-2 is so tightly connected to circadian rhythm and the WCC. It is already known that WCC is able to exert transcriptional control on phospho-OS-2 expression (Lamb *et al.* 2011). It is likely that both the catalytic subunit and the regulatory (RGB-1) subunit of PP2A are able to control expression of the phospho-OS-2 MAPK via the WCC, thus providing additional layers of regulation of phospho-OS-2 MAPK expression.

Recent evidence from *Sordaria macrospora* suggests a role for the *pp2A* homolog SmPP2Ac in regulating cell–cell fusion and sexual development as an integral component of the STRIPAK complex (Bloemendal *et al.* 2012). However, the exact role of SmPP2Ac in control of these developmental outcomes remains to be deciphered. It will be interesting to further investigate whether the PP2A has any role in MAK-2 phosphorylation, a protein that is a major component of cell–cell fusion in *Neurospora* (Fu *et al.* 2011).

Another phosphatase mutant with interesting phenotypes as well as elevated phospho-OS-2 levels in this study is the PP2C class protein phosphatase mutant Δ*pph-8*. When grown on minimal medium, the mutant displayed inappropriate formation of protoperithecia (Figure 3), similar to the S/T kinase mutant Δ*ime-2* (Hutchison *et al.* 2012; Hutchison and Glass 2010). One possible scenario is that IME-2 and PPH-8 regulate two different target phosphoproteins with opposing functions on protoperithecial development. Whereas one target protein could inhibit protoperithecial development on dephosphorylation by PPH-8, the other one could repress it on being phosphorylated by IME-2. Genetic epistasis studies should provide further insight into understanding the underlying mechanism of how *ime-2* and *pph-8* regulate protoperithecial formation in *N. crassa*.

With the success of the *N. crassa* gene knockout project, we have focused on analysis of phenotypes for large groups of genes with crucial roles in cellular homeostasis, including transcription factors, S/T protein kinases, and, now, S/T and tyrosine protein phosphatases. Before our study, most of the protein phosphatases in *N. crassa* had not been characterized. In numerous cases, we now have important clues to their functions. Further studies of these protein phosphatases should provide a greater understanding of how these proteins are able to regulate important cellular roles in *N. crassa*, related fungi, and other eukaryotic organisms.

## Supplementary Material

Supporting Information
