# Supplementary material for: Global Analysis of Serine/Threonine and Tyrosine Protein Phosphatase Catalytic Subunit Genes in Neurospora crassa Reveals Interplay Between Phosphatases and the p38 Mitogen-Activated Protein Kinase
Source: G3 (Bethesda). 2013 Dec 17;4(2):349–65. doi: 10.1534/g3.113.008813 (PMC3931568; doi:10.1534/g3.113.008813)
Supplement: Supporting Information [file supp_4_2_349__index.html]

Global Analysis of Serine/Threonine and Tyrosine Protein Phosphatase Catalytic Subunit Genes in Neurospora crassa Reveals Interplay Between Phosphatases and the p38 Mitogen-Activated Protein Kinase — Supporting Information 

# Global Analysis of Serine/Threonine and Tyrosine Protein Phosphatase Catalytic Subunit Genes in *Neurospora crassa* Reveals Interplay Between Phosphatases and the p38 Mitogen-Activated Protein Kinase

## Supporting Information for Ghosh *et al.*, 2014

**Files in this Data Supplement:**

- Supporting Information - Tables S1-S2 (PDF, 419 KB)
- Table S2 - Primers used during this study. (PDF, 301 KB)
- Table S1 - Detailed Growth and Developmental Phenotypes Data and Detailed Chemical Sensitivity and Nutritional Phenotype Data (.xlsx, 19 KB)
