# Supplementary material for: Global Analysis of Serine/Threonine and Tyrosine Protein Phosphatase Catalytic Subunit Genes in Neurospora crassa Reveals Interplay Between Phosphatases and the p38 Mitogen-Activated Protein Kinase
Source: G3 (Bethesda). 2013 Dec 17;4(2):349–65. doi: 10.1534/g3.113.008813 (PMC3931568; doi:10.1534/g3.113.008813)
Supplement: Supporting Information [file supp_g3.113.008813_008813SI.pdf]

G3 Genes | Genomes | Genetics

Global analysis of serine/threonine and tyrosine protein phosphatase catalytic subunit genes in *Neurospora crassa* reveals interplay between phosphatases and the p38 MAPK

Arit Ghosh[*§†], Jacqueline A. Servin[*†], Gyungsoon Park[*†1] and Katherine A. Borkovich[*§†‡]

[*]Department of Plant Pathology and Microbiology
[§]Graduate Program in Genetics, Genomics and Bioinformatics
[†]Institute for Integrative Genome Biology
University of California
Riverside, California 92521

[1]Current Address:
Plasma Bioscience Research Center
Kwangwoon University,
Seoul, Republic of Korea
[‡]Corresponding Author:
Email: Katherine.Borkovich@ucr.edu
Phone: (951) 827-2753

DOI: 10.1534/g3.113.008813

Table S1 Detailed Growth and Developmental Phenotypes Data and Detailed Chemical Sensitivity and Nutritional Phenotype Data

Available for download as an Excel file at http://www.g3journal.org/lookup/suppl/doi:10.1534/g3.113.008813/-/DC1

Table S2 Primers used during this study

Gene	FGSC Number	Primer designation	Primer sequence (5' to 3')
NCU02496	16654	Forward-diag[1]	GAACTTCCTAGGTTGCTGG
		Reverse-orf[2]	GCAGCAGGTGAGTCTGGTAGTG
		Reverse-out[3]	GCAGATTGCTCGCTCCATTG
		Reverse-diag[4]	ATGAAGTGCTGGTTGGAAGG
NCU06252	14464	Forward-diag	GCTGTGGTGCGAGACTTTG
		Reverse-diag	CACCATGATTCTCTCCGTCG
hph	Not Applicable	Forward-HPH	CGCCCCAGCACTCGTCCGAAG
		Reverse-HPH	GGCATTCATTGTTGACCTCCA

[1]Forward-diag = Sequence upstream of 5' flanking region of knockout cassette
[2]Reverse-orf = Sequence at 5' end of Open Reading Frame (ORF)
[3]Reverse-out = Sequence at upstream of the 5' end of ORF
[4]Reverse-diag = Sequence downstream of 3' flanking region of knockout cassette
