# Supplementary material for: Global Analysis of Serine/Threonine and Tyrosine Protein Phosphatase Catalytic Subunit Genes in Neurospora crassa Reveals Interplay Between Phosphatases and the p38 Mitogen-Activated Protein Kinase
Source: G3 (Bethesda). 2013 Dec 17;4(2):349–65. doi: 10.1534/g3.113.008813 (PMC3931568; doi:10.1534/g3.113.008813)
Supplement: Supporting Information [file supp_g3.113.008813_TableS2.pdf]

Table S2 Primers used during this study

Gene	FGSC Number	Primer designation	Primer sequence (5' to 3')
NCU02496	16654	Forward-diag[1]	GAACTTCCTAGGTTGCTGG
		Reverse-orf[2]	GCAGCAGGTGAGTCTGGTAGTG
		Reverse-out[3]	GCAGATTGCTCGCTCCATTG
		Reverse-diag[4]	ATGAAGTGCTGGTTGGAAGG
NCU06252	14464	Forward-diag	GCTGTGGTGCGAGACTTTG
		Reverse-diag	CACCATGATTCTCTCCGTCG
hph	Not Applicable	Forward-HPH	CGCCCCAGCACTCGTCCGAAG
		Reverse-HPH	GGCATTCATTGTTGACCTCCA

[1]Forward-diag = Sequence upstream of 5' flanking region of knockout cassette
[2]Reverse-orf = Sequence at 5' end of Open Reading Frame (ORF)
[3]Reverse-out = Sequence at upstream of the 5' end of ORF
[4]Reverse-diag = Sequence downstream of 3' flanking region of knockout cassette
